# Supplementary material for: Transcription Factor Deformed Wings Is an Atg8a-Interacting Protein That Regulates Autophagy
Source: Cells. 2024 Nov 17;13(22):1897. doi: 10.3390/cells13221897 (PMC11592666; doi:10.3390/cells13221897)
Supplement: Supplementary file 1 [file cells-13-01897-s001.zip › cells-3272415-supplementary.pdf]

A

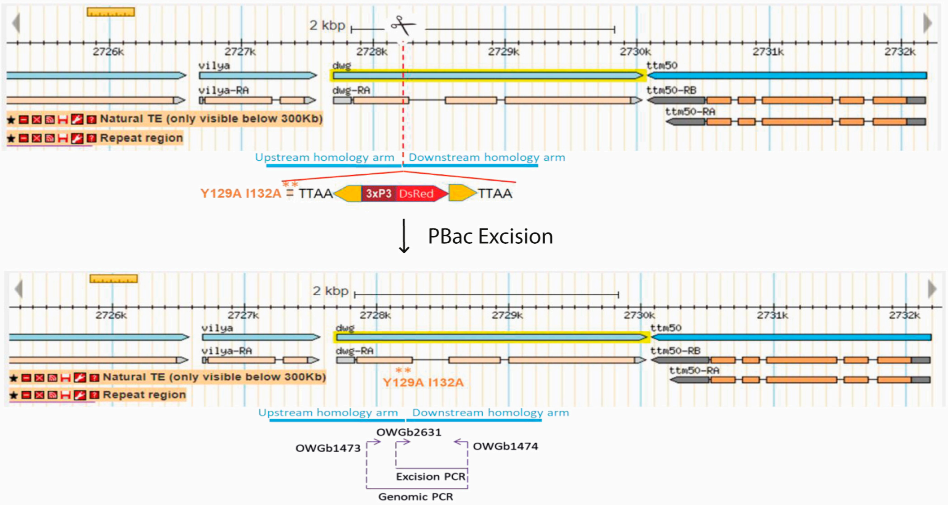

B

220420ex4_OWGb1473 (Sbjct) vs 220420ex excised donor (Query)

420aex4b1473_b1473

Sequence ID: **Query_8363** Length: **567** Number of Matches: **1**

Range 1: 6 to 563 Graphics ▼ Next Match ▲ Pre

Score	Expect	Identities	Gaps	Strand
1009 bits(546)	0.0	555/559(99%)	2/559(0%)	Plus/Plus

```
Query  637   TCGATTCGGACGAGCTGCTCCGCCTGGGCCTGGACGAGGCGCGCTGCTCCAGCTTTCACG  696
             ||||||||||||||||||||||||||||||||||||||||||||||||||||||||||||
Sbjct  6     TCGATTCGGACGAGCTGCTCCGCCTGGGCCTGGACGAGGCGCGCTGCTCCAGCTTTCACG  65

Query  697   AGAGTCAGACTCATAGCCCCAACCAGAGCCAGCAGCACGACCAGCAACAGCAGGCGTTCG  756
             ||||||||||||||||||||||||||||||||||||||||||||||||||||||||||||
Sbjct  66    AGAGTCAGACTCATAGCCCCAACCAGAGCCAGCAGCACGACCAGCAACAGCAGGCGTTCG  125
                         Y129A        I132A
Query  757   ACTCCACCGAAGAGGCCGTCATGGCCGAGATGCTTAACGAGGAGCAGGAGAACGTCGCCA  816
             ||||||||||||||||||||||||||||||||||||||||||||||||||||||||||||
Sbjct  126   ACTCCACCGAAGAGGCCGTCATGGCCGAGATGCTTAACGAGGAGCAGGAGAACGTCGCCA  185

Query  817   ATGTAAGTATCTATCACTTCACGGTGCAATGGAATGCAATGGTTCTTGTCCACTCCCATA  876
             |||||||| |||||||||||||||||||||||||||||||||||||||||||||||||||
Sbjct  186   ATGTAAGTAGCTATCACTTCACGGTGCAATGGAATGCAATGGTTCTTGTCCACTCCCATA  245

Query  877   GAGGATTAACGATTGCTCACAGCGGGCACCAGTCACTCAAATCGATTCACTTATATTGTG  936
             ||||||||||||||||||||||||||||||||||||||||||||||||||||||||||||
Sbjct  246   GAGGATTAACGATTGCTCACAGCGGGCACCAGTCACTCAAATCGATTCACTTATATTGTG  305

Query  937   GCTCTTTGGGATGTAGTAGGGTAGGTCGGTGCCAAAGGAGTGCAACCTGTACCTCCATAC  996
             ||||||||||||||||||||||||||||||||||||||||||||||||||||||||||||
Sbjct  306   GCTCTTTGGGATGTAGTAGGGTAGGTCGGTGCCAAAGGAGTGCAACCTGTACCTCCATAC  365

Query  997   AATGGATAACTTGTCTGCTCTTGGTGACACTACGTCTAAATAGGAGATGGGCTTCATCAA  1056
             |||||||||||||| |||||||||||||||||||||||||||||||||||||||||||||
Sbjct  366   AATGGATAACTTGTATGCTCTTGGTGACACTACGTCTAAATAGGAGATGGGCTTCATCAA  425

Query  1057  GTCTAACTCCAAAGTAAAACCATAACACCTTCTTCATCCGCAGTTGGATGAAGAACTAGC  1116
             ||||||||||||||||||||||||||||||||||||||||||||||||||||||||||||
Sbjct  426   GTCTAACTCCAAAGTAAAACCATAACACCTTCTTCATCCGCAGTTGGATGAAGAACTAGC  485

Query  1117  AGAGGAGGATCGTCGCATCTTCGCGATGACCGTggatgaggaggaggagtttctcaccga  1176
             ||||||||||||||||||||||||||||||||||||||||||||||||||||||||||||
Sbjct  486   AGAGGAGGATCGTCGCATCTTCGCGATGACCGTGGATGAGGAGGAGGAGTTTCTCACCGA  545

Query  1177  ggaggtggatcagg-atga  1194
             ||||||||| |||| ||||
Sbjct  546   GGAGGTGGA-CAGGGATGA  563
```

Supplementary Figure 1
